# Assessment of DNA extraction methods for human gut mycobiome analysis

**DOI:** 10.1098/rsos.231129

**Published:** 2024-01-10

**Authors:** Piyapat Rintarhat, Yong-Joon Cho, Hong Koh, Sowon Park, Eun Joo Lee, Hyeji Lim, Jihye Noh, Dong-Woo Lee, Won Hee Jung

**Affiliations:** ^1^ Department of Systems Biotechnology, Chung-Ang University, Anseong 17546, Korea; ^2^ Department of Molecular Bioscience, Kangwon National University, Chuncheon 24341, Korea; ^3^ Division of Gastroenterology, Hepatology and Nutrition, Department of Pediatrics, Severance Hospital, Yonsei University College of Medicine, Seoul, Korea; ^4^ Department of Biotechnology, Yonsei University, Seoul, Korea

**Keywords:** DNA extraction, fungal community, gut, human faeces, mock community, mycobiome

## Abstract

The gut mycobiome plays an important role in the health and disease of the human gut, but its exact function is still under investigation. While there is a wealth of information available on the bacterial community of the human gut microbiome, research on the fungal community is still relatively limited. In particular, technical methodologies for mycobiome analysis, especially the DNA extraction method for human faecal samples, varied in different studies. In the current study, two commercial kits commonly used in DNA extraction, the QIAamp® Fast DNA Stool Mini Kit and DNeasy PowerSoil Pro Kit, and one manual method, the International Human Microbiome Standards Protocol Q, were compared. Furthermore, the effectiveness of two different bead-beating machines, the Mini-Beadbeater-16 and FastPrep-24^TM^ 5G, was compared in parallel. A mock fungal community with a known composition of fungal strains was also generated and included to compare different DNA extraction methods. Our results suggested that the method using the DNeasy PowerSoil Pro Kit and Mini-Beadbeater-16 provides the best results to extract DNA from human faecal samples. Based on our data, we propose a standard operating procedure for DNA extraction from human faecal samples for mycobiome analysis.

## 1. Introduction

The human gut is colonized by diverse microorganisms, including bacteria, viruses, protozoa and fungi [1–3]. While gut bacteria have received most of the attention in research on the human microbiome due to their abundance [4,5], several studies have suggested that a fungal microbiome (mycobiome) is also found in the human gut. The predominant phylum of fungi that is frequently found in the human gut is Ascomycota, which includes several genera such as *Candida*, *Saccharomyces* and *Aspergillus*. Basidiomycota such as *Penicillium*, *Malassezia,* and *Rhodotorula* are also often observed [6–10].

The gut mycobiome may play an important role in the health and disease of the human gut, although its exact function is still under investigation. Numerous studies reported an altered intestinal fungal community structure in inflammatory bowel disease (IBD) patients and have suggested that this altered mycobiome induces an abnormal immune response, contributing to disease [11]. Indeed, the association of intestinal fungi with IBD has been suggested by several studies. Examples include an increase of anti-*Saccharomyces cerevisiae* antibodies (ASCA), which detect mannan, a carbohydrate constituent of the fungal cell wall, in IBD patients, especially in Crohn's disease [12,13]. Undoubtedly, *Candida albicans* is one of the well-known gut commensal fungi, and studies have suggested an increased abundance of *Ca. albicans* in the faecal samples of IBD patients, although the results varied in different individuals [11]. Interestingly, however, a study also showed a positive correlation between responsiveness of faecal microbiota transplantation (FMT) and the abundance of *Ca. albicans* before FMT treatment [14]. Moreover, a recent study suggested that the CARD9 polymorphism is associated with immune responses to members of the gut fungal mycobiota, such as *Malassezia*, and might play an important role in IBD patients [15].

While there was a wealth of information, such as the optimal protocol for DNA extraction, library preparation and computational analysis of sequencing data, available on the bacterial community of the human gut microbiome, research on the fungal community is still relatively limited. In particular, technical methodologies for mycobiome analysis, especially the DNA extraction method from human faecal samples, varied in different studies and a standard protocol has not yet been established [16–19]. It is critical to obtain as wide a range of fungal taxa as possible. However, fungi have rigid cell walls, which are composed of complex polysaccharides, such as chitin, glucans and mannans, as well as glycoproteins that provide structural support and protection to the fungal cell, making the development of an efficient DNA extraction method, especially from faecal samples, a major challenge [20,21]. It has been suggested that the process of DNA extraction from faecal samples was the most critical factor for microbiome analysis [22–24].

Several previous studies have suggested the effectiveness of DNA extraction methods from faecal samples for microbiome analysis. The International Human Microbiome Standards (IHMS) Project (https://human-microbiome.org/) has provided a method called protocol Q for DNA extraction from human faecal samples [25]. However, the protocol includes multiple steps for extracting DNA and is mainly established for bacterial DNA from faecal samples. Additionally, the IHMS protocol Q can be time-consuming, which may be a disadvantage for high-throughput studies requiring rapid turnaround times. As previously mentioned, mechanical lysis of the fungal cells within the samples is critical because it affects the quality and quantity of the extracted DNA. Bead-beating (BB) is one of the most popular methods to mechanically disrupt fungal cell walls, which is also commonly used to extract DNA from the faecal samples for microbiome analysis [26,27]. A mock community is commonly included as a control or a standard for microbiome analysis [19,28]. However, there is currently no guideline on the appropriate concentration or composition of the mock community, even for bacterial microbiome studies.

Hence, overall, several studies have suggested the importance of investigating the impact of different DNA extraction methods and the need to establish a standard protocol for mycobiome analysis. Huseyin *et al*. studied the impact of five different DNA extraction methods (QIAamp Fast DNA Stool Mini Kit, QIAamp Fast DNA Stool Mini Kit and Bead-beating, QIAamp Fast DNA Stool Mini Kit and Lyticase lysis buffer, FastDNA SPIN Kit, and Repeat bead-beating C [RBBC] column) to analyse the fungal diversity of human faecal samples [16]. While the results of that study provided useful information, of the five methods considered, only two (BB and RBBC) used bead-beating for fungal genomic DNA extraction and demonstrated high yields and quality suitable for library preparation and diversity analysis. Breakdown of the fungal cell wall is a crucial step to extract fungal genomic DNA, and physical disruption using bead-beating showed significant increase of DNA yield compared with other methods such as enzymatic degradation of the cell wall [29–31]. Therefore, a more comprehensive comparison is still required to establish the best method for fungal DNA extraction from human faeces for the mycobiome study.

In the current study, we aimed to propose an effective protocol for DNA extraction from human faecal samples for fungal community analysis. Two commercial kits commonly used in DNA extraction, the QIAamp Fast DNA Stool Mini Kit and DNeasy PowerSoil Pro Kit, and one manual method, the IHMS protocol Q, were compared for the quantity and quality of the extracted DNA, and their influence on fungal diversity analysis. Furthermore, the effectiveness of two different bead-beating machines, the Mini-Beadbeater-16 and FastPrep-24™ 5G, was compared in parallel. Whether different DNA extraction methods influenced fungal diversity analysis by amplicon sequencing was also investigated. Moreover, we generated a mock fungal community with a known composition of fungal strains and included it to compare different DNA extraction methods.

## 2. Material and methods

### 2.1. Sample preparation

Human faeces were obtained at Severance Hospital, Yonsei University College of Medicine, Korea. Faecal samples from six individuals with ulcerative colitis (four females and two males aged between 22 and 45 years) were collected between 4 January and 25 July 2022, and kept at −80°C until use. Total DNA was extracted using the following three methods: the QIAamp Fast DNA Stool Mini Kit (cat. no. 51604; Qiagen, Germantown, MD, USA), DNeasy PowerSoil Pro Kit (cat. no. 47014; Qiagen, Germantown, MD, USA), and IHMS protocol Q by the International Human Microbiome Standard [25]. To compare the effectiveness of the bead-beating machine, two different beating machines, the Mini-Beadbeater-16 (BioSpec, Bartlesville, OK, USA) and FastPrep-24^TM^ 5G (MP Biomedicals, Santa Ana, CA, USA) were used.

### 2.2. DNA extraction and determination of concentration and purity

The DNA extraction was performed as recommend by the manufacturers (QIAamp Fast DNA Stool Mini Kit, DNeasy PowerSoil Pro Kit, and IHMS protocol Q) with slight modifications. Details of the methods are described in the electronic supplementary material. The concentration of the extracted DNA was determined using the NanoDrop ND-1000 (Thermo Fisher Scientific, Waltham, MA, USA) and the DNA purity was assessed by measuring the absorbance ratio at 260/280 nm. The extracted DNA was kept at −80°C until use.

### 2.3. Spike preparation

*Cryptococcus neoformans* H99 was used as a spike fungal cell [32]. The fungus was cultured on yeast peptone dextrose (YPD) agar (10 g yeast extract, 20 g bacto peptone, 20 g bacto agar and 40% glucose per litre) at 30°C for 2 days. A single colony was inoculated in 3 ml liquid YPD and grown at 30°C for 24 h. One millilitre of cells was harvested, washed twice with phosphate-buffered saline (PBS), and resuspended in YPD to adjust the concentration for use as a spike. A total of 100 µl containing 10^3^ *Cr. neoformans* H99 cells was added to 0.25 g of each faecal sample. To ensure that the faeces and spiked fungal cells were thoroughly mixed, the sample was homogenized using the vortex mixer at maximum speed for 10 min. The spike-in faecal samples were kept at −80°C until use.

### 2.4. Quantitative real-time polymerase chain reaction

Quantitative real-time polymerase chain reaction (Q-PCR) was performed using the Bio-Rad CFX Connect Real-Time PCR System (Bio-Rad, Hercules, CA, USA). The primers specific for *Cr. neoformans* H99, CTR1_F (5'-GGTACAATAGGAGGTGACCGT-3') and CTR1_R (5-'CCGAAGATGGCATCCAAGATG-3'), were designed to assess the levels of the spiked fungal cells in the extracted DNA. The extracted genomic DNA from the pure culture of *Cr. neoformans* H99 was used as the positive control. The primers, ITS4 (5'-TCCTCCGCTTATTGATATGC-3') and ITS5 (5'-GGAAGTAAAAGTCGTAACAAGG-3'), were used to amplify the fungal internal transcribed spacer (ITS) region in the samples [33]. Each Q-PCR reaction contained 10 µl of AccuPower*®* 2X GreenStar qPCR Master Mix (Bioneer, Daejeon, Korea), 0.5 pg–100 ng of DNA template, 2 µl of forward and reverse primers, and was filled with sterilized water to 20 µl. The following amplification programme was used: 40 cycles of 15 min at 95°C and 15 sec at 95°C, followed by 30 sec at 52°C.

### 2.5. Mock community

To evaluate the effectiveness of different DNA extraction methods, we generated a mock community in which the fungal members were chosen based on availability of their reference genome sequences [34]. The following six fungal species were used. *Candida albicans* SC5314, *Ca. glabrata* CBS138 and *S. cerevisiae* BY4742 were grown on YPD agar at 30°C for 2 days and then cultured in liquid YPD at 30°C with overnight shaking at 200 r.p.m. *Malassezia restricta* KCTC27527 and *M. furfur* CBS7966 were grown on Leeming and Notman agar (LNA) at 34°C for 3 days and then cultured in 10 ml liquid mDixon (36 g malt extract, 20 g bile salts, 6 g peptone, 10 ml Tween 40, 4 ml 50% glycerol, 2 ml oleic acid and 15 g Bacto agar per litre, pH 6.8) at 34°C with 3 days shaking at 200 r.p.m. *Aspergillus fumigatus* Af293 was grown on Czapek Dox agar (CZA) and incubated at 30°C for 5–7 days. Using a sterile swab, spores of *A. fumigatus* were taken from the CZA agar plate and suspended in 10 ml of sterile PBS (pH 7.3) containing 0.05% v/v Tween 20 and incubated at room temperature for 30 min to enable the hyphae to settle at the bottom to only transfer the upper phase for cell counting [19]. Fungal cells were counted using a haemocytometer, and the cells were adjusted at the same concentration ratio. In this study, the mock community was divided into groups for two separate sets of DNA extraction. In the first set, 100 µl of an equal number of each fungal species (final concentration of 6 × 10^6^ cells for each species) were directly added into 0.25 g of faeces in a 2 ml microtube and the sample was homogenized using the vortex mixer at maximum speed for 20 min. The second set contained only the fungal cells without faeces.

### 2.6. Library construction and sequencing

The sequencing libraries were prepared according to the Illumina Metagenomic Sequencing Library protocols to amplify the ITS3 and ITS4 regions. The input gDNA (10 ng) was PCR-amplified with the 5 × reaction buffer, 1 mM of dNTP mix, 500 nM each of the universal F/R PCR primers, and the Herculase II fusion DNA polymerase (Agilent Technologies, Santa Clara, CA, USA). The cycle condition for the first PCR was 3 min at 95°C for heat activation, and 35 cycles of 30 s at 95°C, 30 s at 55°C and 30 s at 72°C, followed by a 5 min final extension at 72°C. The universal primer pair with the Illumina adapter overhang sequences used for the first amplifications were as follows:

ITS3 Amplicon PCR Forward Primer:

5'-TCGTCGGCAGCGTCAGATGTGTATAAGAGACAGGCATCGATGAAGAACGCAGC-3′

ITS4 Amplicon PCR Reverse Primer:

5′-GTCTCGTGGGCTCGGAGATGTGTATAAGAGACAGTCCTCCGCTTATTGATATGC-3′

The first PCR product was purified with AMPure beads (Agencourt Bioscience, Beverly, MA, USA). Following purification, 10 µl of the first PCR product was PCR amplified for final library construction containing an index using the Nextera XT Indexed Primer. The cycle condition for the second PCR was the same as the first PCR condition except for 10 cycles. The PCR product was purified with AMPure beads. The final purified product was then quantified using qPCR according to the qPCR Quantification Protocol Guide (KAPA Library Quantification kits for Illumina Sequencing platforms) and qualified using the TapeStation D1000 ScreenTape (Agilent Technologies, Waldbronn, Germany). The product was then sequenced using the MiSeq platform (Illumina, San Diego, USA).

### 2.7. Mycobiome data analysis

Generated raw reads were trimmed based on the quality and the adapters were removed using Trimmomatic v. 0.36 [35]. The cleaned paired-end reads were merged using PEAR v. 0.9.6 [36] and the results were imported to the QIIME 2 pipeline v. 2020.8 [37]. Taxonomic assignment was performed using the Targeted Host-associated Fungi (THF) v. 1.6.1 mycobiome database [38]. α-diversity and β-diversity metrics were measured in QIIME 2 using the default parameters.

### 2.8. Statistical analysis

The statistical differences in DNA yield between the various DNA extraction methods were compared using the Q-PCR threshold cycle (Ct) values of each sample to calculate the relative quantification (RQ). Moreover, the unpaired t-test analysis was made using the Prism GraphPad program by Dotmatics and the *p*-values between each group were calculated to compare the difference in fungal DNA yields between the groups. The statistical significance was defined as a *p*-value ≤ 0.05.

## 3. Results

### 3.1. Comparisons of different DNA extraction methods for fungal community analysis in faeces

Multiple studies demonstrated that different DNA extraction methods caused bias and significantly influenced the analysis of the bacterial communities by sequencing [22]. In this study, three different DNA extraction methods, the QIAamp Fast DNA Stool Mini Kit, DNeasy PowerSoil Pro Kit and IHMS protocol Q (hereafter named the QIAstool, DNeasy and IHMS methods, respectively), were compared to establish the best method for fungal community analysis in human faecal samples. Moreover, two different bead-beating machines, the Mini-Beadbeater-16 and FastPrep-24^TM^ 5G (hereafter named Mini-beadbeater and FastPrep, respectively), were used and their efficiency was compared since disruption of the fungal cell membrane was critical for DNA extraction from fungi.

Faeces from six individuals with ulcerative colitis were used, and *Cr. neoformans*, which is not frequently observed in human faeces, was added as a spike to each faecal sample prior to DNA extraction to evaluate the efficiency of the three different DNA extraction methods using two different bead beaters. The spike-in method aims to add a known cell concentration directly into the faecal sample so that the extracted DNA from the spike in each DNA extraction method can be compared by calculating the initial concentration and the final concentration after the DNA extraction process [18]. After the DNA was extracted from the faecal samples, yield, purity and processing time were determined. Among the methods, the IHMS generally showed the highest yield, while the purity of the extracted DNA was similar to those of the other methods tested. Regarding the processing time, the DNeasy method required the shortest processing time to complete the extraction (table 1).

**Table 1.** Concentrations, purity and processing time of different DNA extraction methods tested in the current study.

method	bead beater	concentration (µg µl^−1^)	purity (A260/280)	time per sample (min)
QIAstool	Mini-Beadbeater-16 (BioSpec)	0.25 ± 0.09	2.03 ± 0.09	57
DNeasy		0.05 ± 0.02	1.87 ± 0.08	42
IHMS		0.56 ± 0.33	2.04 ± 0.06	196
QIAstool	FastPrep-24^TM^ 5G (MP)	0.19 ± 0.07	2.02 ± 0.09	57
DNeasy		0.03 ± 0.02	1.83 ± 0.16	42
IHMS		0.61 ± 0.41	2.06 ± 0.07	196

**Figure 1.**
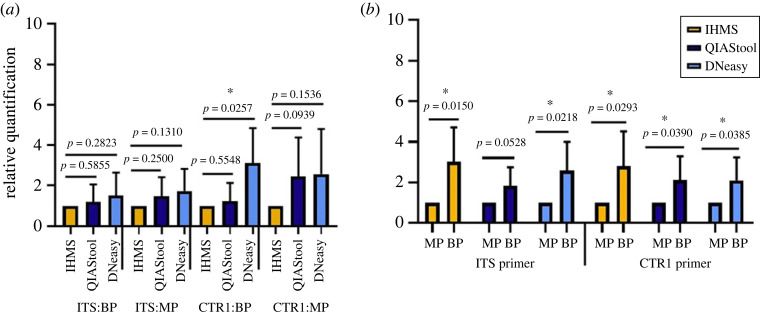
Comparison of fungal DNA levels by Q-PCR. (*a*) Fungal-specific DNAs within the total DNA extracted with different extraction methods were compared using the *CTR1* primers (CTR1) and ITS2 primers (internal transcribed spacer (ITS)) that specifically bind to the genomic DNA of the spiked *Cr. neoformans* and the ribosomal region of every fungal cell in the sample, respectively. (*b*) The same data were reanalysed based on which bead beater was used. MP: FastPrep; BP: Mini-beadbeater. Data are averages from four replicates with error bars indicating standard deviation.

Undoubtedly, DNA from multiple cells such as the host intestine and gut-residing bacteria were included in the extracts from the faecal samples. To estimate fungal-specific DNAs within the whole extracts, we first determined the levels of the fungal DNA by Q-PCR using the primer sets that specifically bound to the *CTR1* gene encoding the copper transporter in the genomic DNA of the spiked *Cr. neoformans* cells [39]. The results of the Q-PCR using the *CTR1* primers showed the highest levels of the spiked fungal DNA in the sample extracted using DNeasy. Similarly, Q-PCR using the fungal-specific ITS primers also showed the highest yield of fungal DNAs in the samples prepared with DNeasy compared with the other extraction methods. Although overall Q-PCR data showed low statistical significance, the result using the DNeasy method with the Mini-beadbeater was statistically significant (*p* = 0.025). Therefore, we concluded that the DNeasy method provides the highest yield of fungal-specific DNA from human faecal samples (figure 1). When we compared the performance of the two different bead beaters, the Mini-beadbeater generated a higher yield of fungal DNA than that of the FastPrep (figure 1*b*). Overall, regarding the yield of the fungal DNA and processing time, our study demonstrated that the DNeasy method provides the best performance.

### 3.2. Amplicon sequencing analysis of DNA extracted with different methods from faecal samples

**Figure 2.**
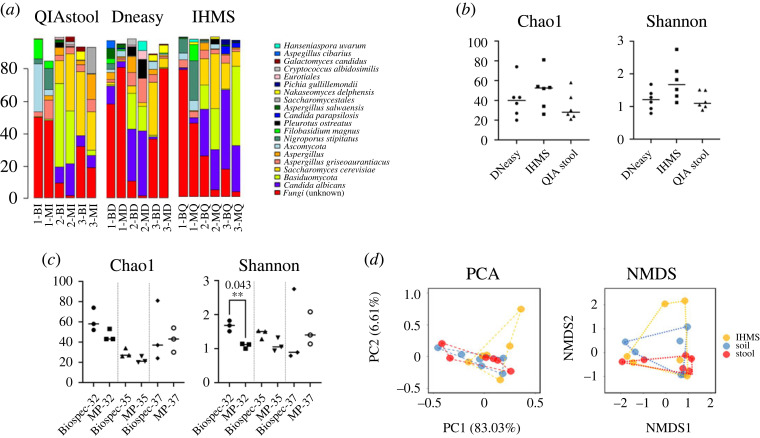
The results of the fungal community analysis. (*a*) Fungal community structures in DNA extracted from faecal samples using three different extraction methods are shown. 1, 2, and 3: faecal samples from three independent individuals. B: Mini-beadbeater; M: FastPrep; I: International Human Microbiome Standard (IHMS); D: DNeasy; Q: QIA-stool. Only the top 20 of all detected species are shown in the figure. (*b*) Comparison of the α-diversity of the samples prepared with different DNA extraction methods. (*c*) Comparison of the α-diversity of the samples prepared with different bead-beating methods. (*d*) Comparison of the β-diversity of the samples prepared with different DNA extraction methods. PCA: principal components analysis; NMDS: non-metric multi-dimensional scaling.

We next investigated how different DNA extraction methods influence the results of the mycobiome analysis for human faecal samples by amplicon sequencing. DNA was extracted from the faeces of three patients with ulcerative colitis using three different methods, the QIAstool, DNeasy and IHMS, with two different bead beaters, the Mini-beadbeater and FastPrep, and fungal communities within the samples were analysed by amplifying the fungal ITS2 region and sequencing on the Illumina MiSeq platform. The 18 pooled samples (three faecal samples; three DNA extraction methods; two bead beaters) returned a total of 1 575 511 ITS2 sequence reads after raw sequence filtration and chimera removal. These reads (ranging from 68 230 to 114 118 per sample) were distributed into 44 fungal taxa assigned at the genus level and 62 taxa assigned at the species level (figure 2*a*). The complete list of the observed taxa is shown in electronic supplementary material, table S1.

**Figure 3.**
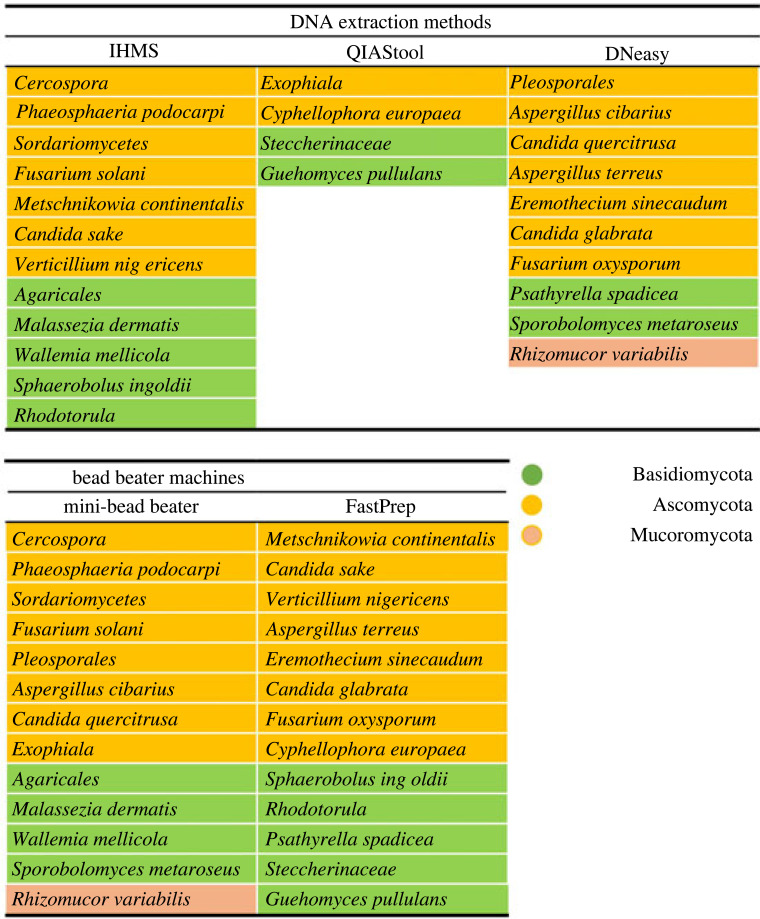
The unique taxa found in each sample prepared with different DNA extraction methods. Observed unique taxa are listed based on different DNA extraction methods (upper panel) and different bead-beating machines (lower panel).

Comparison of the α-diversity showed no significant difference between the different DNA extraction protocols for the three faecal samples. However, we should note that there was a tendency for the IHMS method to provide a higher α-diversity compared with the other two methods, although the results were not statistically significant and variations between the faecal samples were unacceptably high (figure 2*b*). By contrast, when we compared the diversity between the different bead beaters, our results indicated that the sample prepared by the Mini-beadbeater displayed a significantly higher α-diversity than that of the FastPrep for at least one faecal sample (figure 2*c*). Regarding the β-diversity, no statistically significant differences between the groups were noticed when we performed a permutational multivariate analysis of variance (PERMANOVA) (figure 2*d*). The number of unique taxa found in each sample prepared with different DNA extraction methods was also compared. A total of 12 and 10 unique species were found in the DNA samples prepared with the IHMS and DNeasy methods, respectively, while four species were observed in the DNA sample extracted with the QIAstool method. Moreover, only the DNeasy method resulted in finding the fungal species belonging to Mucoromycota. The same number of unique taxa was found when the DNA samples extracted with two different bead beaters were compared. However, only the DNA extracted using the Mini-beadbeater showed the fungus belonging to Mucoromycota (figure 3). Together our data suggested that the three DNA extraction methods displayed no statistically significant difference in efficiency. However, the IHMS and DNeasy methods showed the higher number of unique taxa compared with the QIAstool method. Regarding bead beaters, the Mini-beadbeater showed a higher α-diversity and number of unique taxa than the FastPrep.

### 3.3. A fungal mock community analysis

**Figure 4.**
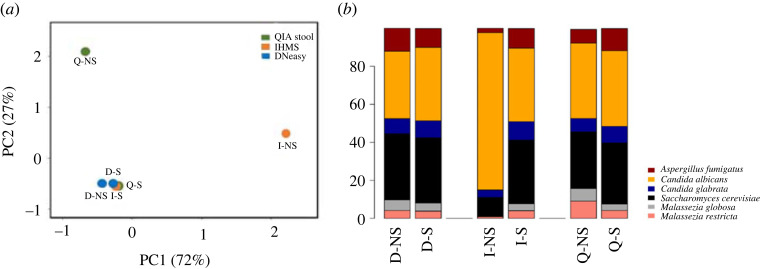
The similarities of the microbial community compositions between samples prepared using different DNA extraction methods were compared. (*a*) The results of the principal component analysis (PCA) are shown. D-S: DNeasy, the mock community in faeces; Q-S: QIAStool, the mock community in faeces; I-S: International Human Microbiome Standard (IHMS), the mock community in faeces; D-NS: DNeasy, the mock community without faeces; Q-NS: QIA-Stool, the mock community without faeces; I-NS: IHMS, the mock community without faeces. (*b*) A relative abundance of each species was also presented in bar graphs. Only the top 20 of all detected species are shown in the figure.

**Figure 5.**
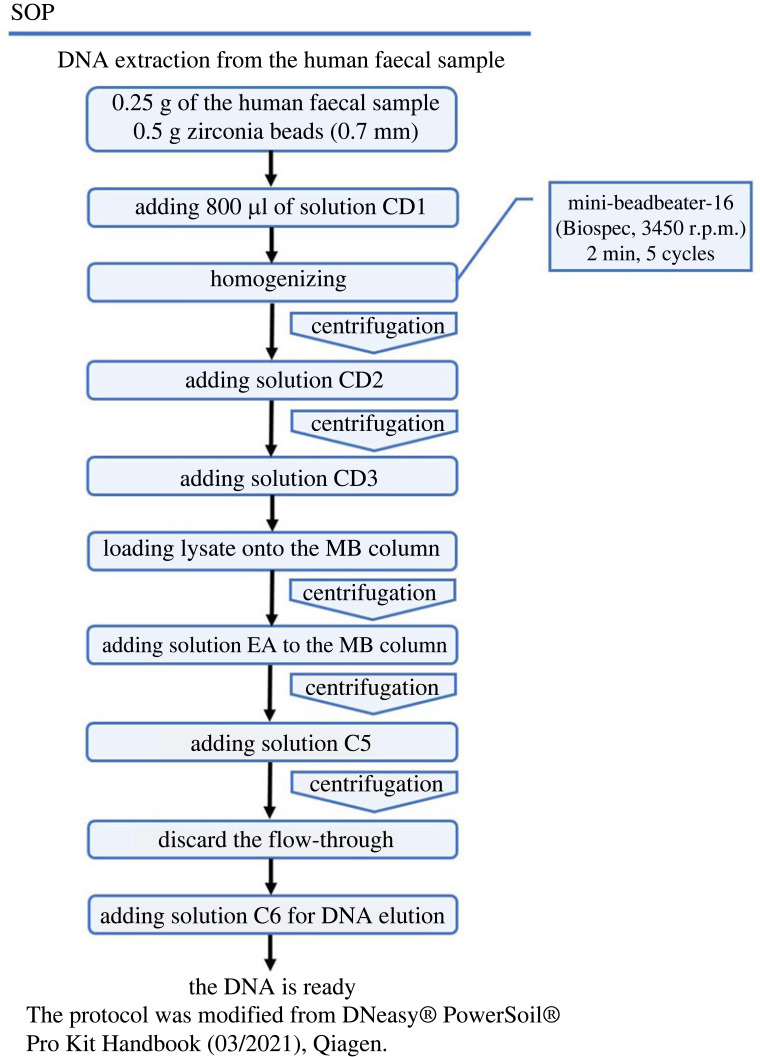
A proposed standard operating procedure (SOP) for DNA extraction from human faeces. The protocol was modified from the DNeasy PowerSoil Pro Kit Handbook (03/2021), Qiagen Inc. (Germantown, MD, USA).

To evaluate the efficiency of different DNA extraction methods in more detail, we developed a fungal mock community by mixing a known number of fungal cells of different species that were frequently identified in human faeces. A total of six different fungal species of which genome sequences were available, *Ca. albicans* SC5314, *Ca. glabrata* CBS138, *S. cerevisiae* BY4742, *A. fumigatus* AF293, *M. restricta* KCTC27527 and *M. globosa* CBS7966 were included. Two separate samples containing mock communities were prepared. One sample contained an equal number of each fungal strain that was mixed with a faecal sample, and the other contained only the fungal cells without faeces. The strategy we chose was to add intact live fungal cells directly to the faeces rather than mixing a known amount of genomic DNA of the selected fungal species, because the purpose of our study included comparing the efficiency of different DNA extraction methods using faecal samples. The total DNA from these samples was extracted using the three different methods described above with cell disruption using the Mini-beadbeater, and the composition of the mock fungal community was analysed by ITS2 amplicon sequencing on the Illumina MiSeq platform. The resulting fungal community profiles of DNA extracted from the sample, of which a mock community was added to the faeces, was compared with that of DNA extracted from the sample containing fungal cells without faeces and extracted by the same methods. We assumed that, ideally, the resulting fungal community profiles for the samples containing the mock community in the faeces should be similar to those without faeces. Therefore, we sought which DNA extraction method provided the highest similarity between the fungal community profiles of the mock community in the faeces and the community without faeces. To determine the differences among the methods, we employed principal component analysis (PCA), a multivariate analysis technique used to investigate the similarities and differences in the composition of the microbial communities between samples [40]. The results showed that the DNeasy method provided the closest correlation between the samples with or without faeces (figure 4) and suggested that DNA extracted by the DNeasy method most closely represented the fungal community structure within the faecal sample compared with the two other methods used in our study. Taken together, the results of our study suggested that the method using DNeasy and the Mini-beadbeater provides the best results to extract DNA from human faecal samples. Based on our results, we propose a standard operating procedure for DNA extraction from human faecal samples for mycobiome analysis (figure 5).

## 4. Discussion

The selection of appropriate DNA extraction methods is crucial for microbiome studies, as it can significantly impact the quality and quantity of microbial DNA in the samples [22,23]. Human faecal samples, in particular, pose challenges for DNA extraction due to the complex physical and chemical properties of faeces [41], which can interfere with the DNA extraction process and lead to reduced yields of high-quality DNA. Several studies compared different DNA extraction methods and reported their efficiencies to propose the most suitable method for mycobiome analysis. However, these studies only compared different DNA extraction methods without comparison of different bead-beating processes, as done in the current study [16–19]. Among them, a recent study by Shaffer *et al*. compared five different DNA extraction protocols for bacterial microbiome and mycobiome analysis in a high-throughput manner. Although the study thoroughly compared different protocols, the experiments were performed in a 96-well format with no particular step for disrupting the fungal cell walls, and therefore, the efficiency of the fungal-specific DNA was not warranted [42]. Nevertheless, the study also showed a higher performance than other methods, as we found in the current study.

In our study, we aimed to propose an efficient standard method for analysing the fungal community in human faecal samples. Three of the most widely used DNA extraction methods for gut microbiome analysis were compared for the quantity and quality of the extracted DNA, and whether this influenced the diversity of the fungal community after sequencing analysis. Among the different methods, the IHMS method demonstrated the highest total DNA yield, as observed in a comparative analysis with other DNA extraction methods. Our results are in concurrence with previous published studies, which also showed that the IHMS method consistently provides high total DNA yields [18,22,28]. We should note that faeces contain multiple cell types, and therefore, the higher total DNA yield does not guarantee obtaining a higher fungal DNA yield from faecal samples for gut mycobiome analysis. In addition, the IHMS method is associated with a relatively longer processing time, which may be a significant factor to consider, especially for a high-throughput study.

To access the yield of fungal DNA from the faecal samples more specifically, we added *Cr. neoformans* as a spike directly to the faecal sample, extracted DNA using different extraction methods, and relatively compared the fungal DNA yield using Q-PCR. *Cryptococcus neoformans* was chosen because data generated by another group and our own unpublished data showed this fungus is rarely found in human faeces, and, therefore, could eliminate any possible bias caused by the presence of the fungus in the faecal samples. The results of the Q-PCR using two independent primer sets, the ITS primers and the *Cr. neoformans*-specific primer, *CTR1*, showed a relatively higher concentration of fungal-specific DNA prepared using the DNeasy method compared with that of the other methods. Data using each primer set showed low statistical significance. However, both results using two independent primers, one for the entire fungal population, and the other for the spiked fungal cells, similarly indicated superiority of the DNeasy method over other methods. The difference in yields could also be explained by the bead-beating process during DNA extraction, which disrupts the fungal cell walls in the samples. Therefore, in the current study, we conducted a comparative analysis of two different bead-beating machines, and found that the Mini-beadbeater generated a higher fungal DNA yield compared with the FastPrep, which has been widely used in several published papers on mycobiome studies [28,43–46].

In addition, to compare the DNA concentration, the influence of different DNA extraction methods on the fungal community analysis was investigated using amplicon sequencing with the ITS2 primers. The ITS primers and 18S rRNA gene primers are commonly employed for amplifying fungal DNA regions [47,48]. The ITS regions are considered more suitable for studies aiming to capture a broad range of fungal taxa and/or require species-level identification, including both filamentous fungi and yeasts [47,49–51]. Previous studies suggested that the use of ITS primers in amplicon sequencing is that the ITS is a more reliable biomarker for fungal diversity due to its greater taxonomic resolution and selective constraint compared with the 18S rRNA [52–54]. However, it should be noted that the selection of different ITS primers could potentially introduce taxonomic biases, and some of the commonly used primers, such as ITS-1F, may contain a high number of mismatches relative to the target sequences [55]. Moreover, some primers, such as ITS1-F, ITS1 and ITS5, have been shown to be biased towards amplification of basidiomycetes [49]. Therefore, in the current study, we used the primers that amplify the ITS2 region, which have been shown to cause fewer biases during PCR amplification and nucleotide sequencing analysis, and that were generally recommended for mycobiome analysis [56,57]

The α-diversity results, which describe the richness of species diversity in a defined ecological community, and β-diversity, which quantitatively compares the overall taxonomic differences between two communities [58,59], showed no statistical significance between the DNA samples prepared using three different DNA extraction methods. However, our results demonstrated that, regarding bead-beating methods, the Mini-beadbeater provided a significantly higher α-diversity compared with the FastPrep. Moreover, we found that the Mini-beadbeater generated a higher number of unique taxa than the FastPrep.

In our study, a fungal mock community, which was composed of six fungal species previously identified in human faeces, was prepared to evaluate the effectiveness of different DNA extraction methods. Our results showed that the DNeasy method displayed a closer correlation between the samples of the mock communities with or without faeces compared with the other methods. This result suggests that the DNeasy method tested in our study provided results that most effectively represent a fungal community. Finally, based on our comprehensive analysis and comparison of different DNA extraction methods, we concluded that the DNeasy method combined with the Mini-beadbeater is the most effective method to extract DNA from human faecal samples for mycobiome analysis.

## Data Availability

The ITS2 sequence reads are available in the NCBI SRA under the BioProject accession numbers PRJNA1002476 and PRJNA1002477 for comparison of the DNA extraction methods and the fungal mock community, respectively. Supplementary material is available online [60].
